# Adsorption and degradation of imazapic in soils under different environmental conditions

**DOI:** 10.1371/journal.pone.0219462

**Published:** 2019-07-08

**Authors:** Wangcang Su, Hongdan Hao, Mingzhen Ding, Renhai Wu, Hongle Xu, Fei Xue, Changchao Shen, Lanlan Sun, Chuantao Lu

**Affiliations:** 1 Henan Key Laboratory of Crop Pest Control, Key Laboratory of Integrated Pest Management on Crops in Southern Region of North China, Institute of Plant Protection, Henan Academy of Agricultural Sciences, Zhengzhou, PR, China; 2 Jinling College, Nanjing University, Nanjing, PR, China; Centre for Ecology and Hydrology, UNITED KINGDOM

## Abstract

Imazapic is widely used in peanut production, and its residues can cause damage to succeeding crops planted in the following year. The planting area of peanut is large in Henan province. Inceptisol is the main soil type in Henan Province and was used in laboratory experiments that were conducted to investigate imazapic degradation in soil under various environmental conditions. The results indicated that the imazapic degradation rate increased with an increase in temperature, soil pH, and soil moisture, and decreased with organic matter content. The use of biogas slurry as a soil amendment accelerated imazapic degradation. The half-life of imazapic in sterilized soil (364.7 d) was longer than in unsterilized soil (138.6 d), which suggested that there was a significant microbial contribution to imazapic degradation. Imazapic adsorption was also examined and was found to be well described by the Freundlich isotherm. The results indicate that soil has a certain adsorption capacity for imazapic.

## Introduction

**Fig 1:**
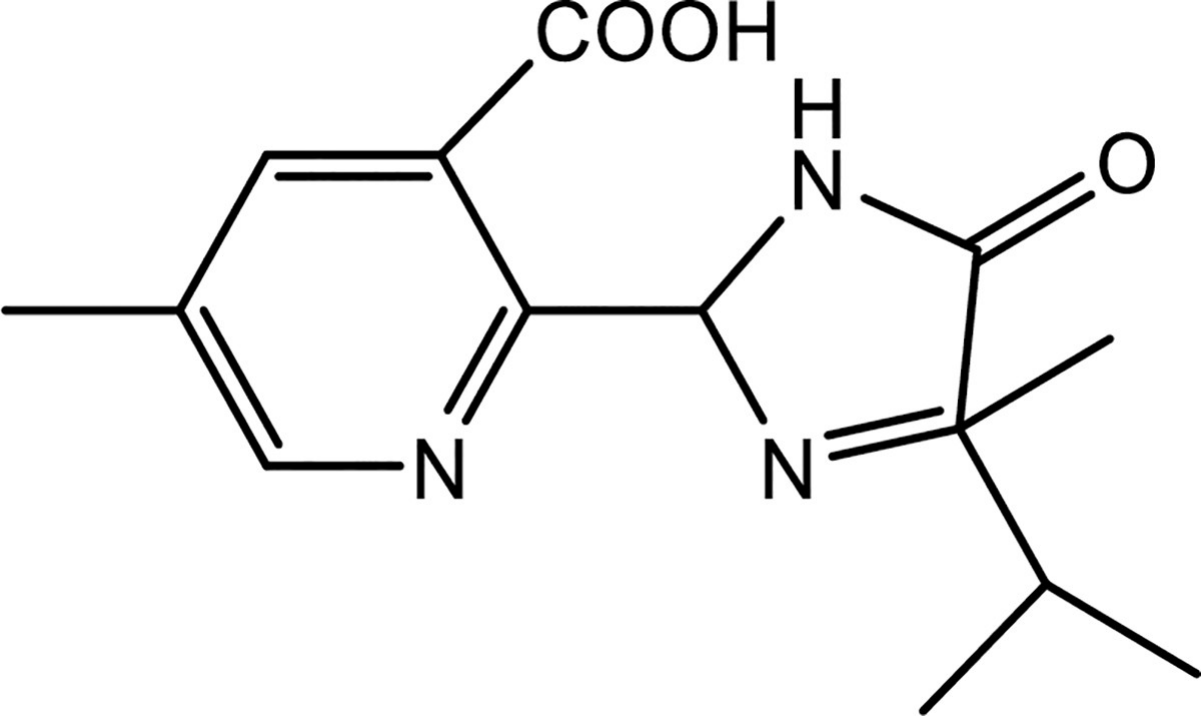
The chemical structure of imazapic.

Imazapic(±)-2-(4-isopropyl-4-methyl-5-oxo-2-imidazolin-2-yl)-5- methylnicotinic acid is an imidazolinone herbicide. Its chemical structure is shown in Fig 1. It can control many common weeds such as lambsquarter (*Chenopodium album L*.) and pigweed species (*Amaranthus* spp.), and troublesome weeds such as yellow nutsedge (*Cyperus esculentus L*.) and purple nutsedge (*Cyperus rotundus* L.) [1–3]. Because of its tolerance by peanut (*Arachis hypogaea* L.), as well as its high-efficiency and broad-spectrum application, imazapic has been widely used in peanut cultivation worldwide. Imazapic is registered for use in peanut and sugar cane (*Saccharum officinarum*) production in China [4,5]. The planting area of peanut in China was 4,616,000 hectares in 2015 according to statistics from the National Bureau of Statistics of China [6]. The planting area of peanut is large in Henan province, and it was reported that Henan province had the largest planting area of peanuts in China in 2005 [7].

Imazapic is highly effective, with a low toxicity, but has a high soil persistence due to its slow degradation [8,9]. The half-life of imazapic has been reported to be 233 d in the field [10,11]. According to Marchesan [12], imidazolinone herbicides can remain in the soil for up to two years after application. York et al. reported that imazapic delayed cotton maturity and reduced the yield by 44% the year after application in peanuts (140 g ai ha^−1^) [4]. The imazapic product label (PD20070370, PD20150487) clearly indicates that cultivation of the following crops requires a safety interval after application: wheat (4 months); cotton, corn and barley (18 months); and rape, cucumber, and spinach (24 months) [13,14].

Imidazolinone herbicides are reported to be primarily degraded by microbes, and this is directly associated with soil conditions and soil adsorption [13,15]. The adsorption and degradation of pesticides are coupled processes, and are affected by various environmental conditions (e.g., temperature, soil moisture, soil pH, and organic matter content). However, most previous research on imazapic has focused on its effects on succeeding crops, and the effect of environmental factors on imazapic degradation has not been thoroughly examined. The objective of this study was: (1) to investigate the degradation of imazapic in the laboratory under various environmental conditions, including temperature, soil moisture, soil pH, sterilized and unsterilized soil, and the effect of chicken manure and biogas slurry as a soil amendment. (2) We also examined imazapic adsorption in soil. Understanding the degradation of imazapic under various environmental conditions will result in a better prediction of its environmental behavior and provide a useful reference for safety evaluation.

## Materials and methods

### Soils and materials

The soil used in the study was topsoil (0–10 cm) collected from a crop field at Xinxiang (113°48'E, 35°27'N), Henan Province, China, with no history of imazapic application. According to the United States Department of Agriculture soil taxonomy the soil is classified as an Inceptisol (Cambisols, according to the World Reference Base for Soil Resources nomenclature). Inceptisol is the main soil type in Henan Province [16]. The properties of the soil are presented in Table 1. The soil used in the laboratory was air-dried and sieved through 2-mm sieves prior to use. Chicken manure was applied as a source of organic matter in the study and was purchased from a chicken farm in Kaifeng (China) and then air-dried, homogenized, and sieved through 2-mm sieves. Biogas slurry was collected from a household biogas digester, with the main material being pig manure, sieved through 2-mm sieves prior to use. The characteristics of chicken manure and biogas slurry are presented in Table 2. Imazapic standards (98.0% purity) were purchased from Dr. Ehrenstorfer GmbH (Augsburg, Germany). HPLC-grade methanol was purchased from Merck (Darmstadt, Germany). Formic acid (96.0%) was purchased from TEDIA (Fairfield, OH, USA). Analytical reagent grade sodium hydroxide (NaOH), phosphoric acid (98%), hydrochloric acid (HCl) (37%), and trichloromethane were purchased from Sinopharm Chemical Reagent Co., Ltd. (Beijing, China). Syringe filters (PTFE, 0.22 μm) were obtained from Supelco (Bellefonte, PA, USA). Imazapic (1000 mg ae/L) was prepared in methanol, and the stock standard solution was diluted as required.

**Table 1:** Properties of the soil.

Location (Soil type)	pH	Organic matter (%)	Sand (%)	Slit (%)	Clay (%)	Quartz (%)	Feldspar (%)	Vermiculite (%)
Xinxiang (Inceptisol)	8.1	0.55	21.8	69.1	9.1	26	17	11

**Table 2:** The characteristics of the soil amendment.

Materials	pH	Organic matter (%)
Chicken manure	7.1	21.5
Biogas slurry	7.0	20.1

### Adsorption experiments

Imazapic adsorption was studied in a batch equilibrium system, which was conducted in the laboratory at room temperature (approximately 25°C) [17]. A preliminary study was conducted to determine the ratio of solution and soil. The tested soil:solution (Wsoil:Vsolution) ratios were 1:2, 1:5, and 1:10. With a soil:solution ratio of 1:5 adsorption was too high, while it was too low at a soil:solution ratio of 1:10. The 1:5 soil:solution ratio was selected to study the adsorption of imazapic. For the determination of adsorption equilibration time, each soil sample (5 g of dry weight equivalent) was collected in centrifuge tubes, to which 25 mL of 0.5 mg/L imazapic solution was added (prepared in 0.01 M CaCl_2_ solution). Tubes were shaken for different times (2, 4, 6, 8, 10, 12, 18, and 24 h) and centrifuged for 5 min at a relative centrifugal force (RCF) of 1615 × g (3800 r/min). Equilibrium was achieved after 12 h of shaking. Tubes containing 25 mL of 0.5 mg/L imazapic solution (prepared in 0.01 M CaCl_2_ solution) without soil served as control samples. For adsorption isotherm studies, each soil sample (5 g of dry weight equivalent) was transferred to 100-mL centrifuge tubes, to which 25 mL (0.02, 0.04, 0.06, 0.08, 0.2 and 0.5 mg/L) imazapic solution was added (prepared in 0.01 M CaCl_2_ solution). The maximum methanol concentration in the used solutions was 0.05%. Tubes were then continuously shaken and centrifuged for 5 min at an RCF of 1615 × g (3800 r/min) at the end of the equilibrium period (after 12 h of shaking). A 10 mL portion of the supernatant from each tube was withdrawn carefully without soil and transferred into a 200-mL separatory funnel, and the supernatant pH value was adjusted to 1.8–2.0 by adding HCl (7 mol/L). The organic phase was collected after three successive extractions using 20 mL of trichloromethane, and the combined organic phase was concentrated by a rotary evaporator at 40°C. The residue was dissolved in methanol, with a final volume of 2 mL, filtered through 0.22-μm syringe filters, and transferred into an auto-sampler vial for analysis. The amount of imazapic adsorbed was calculated from imazapic concentrations in the initial aqueous solution (before equilibrium) and the concentrations in the supernatant (after adsorption equilibration). For each imazapic concentration, there were three replicates.

The adsorption ratio A was calculated as follows:

$$A=\frac{M-C_{e}\times V_{0}}{M}\times 100$$

Here, *A* is the adsorption ratio, %; *M* is the quantity of imazapic in the stock solution used in the adsorption study, μg; *Ce* is the concentration of imazapic in the supernatant at the equilibration time, g/mL; and *V0* is the volume of stock solution used in the adsorption study, mL.

The Freundlich equation is as follows:

$$Cs={Κ}_{f}\times {\left(Ce\right)}^{1/n}$$

Here, Cs is the amount of imazapic adsorbed by the soil, mg/g; Ce is the concentration of imazapic in the supernatant at equilibration time, mg/L; and K_f_ and 1/n are Freundlich’s adsorption coefficient and the adsorption constant, respectively.

### Degradation experiment

#### Soil treatment and sampling

Each treatment consisted of 1000 g of soil dry weight equivalent, with 20 mL of 50 mg/L imazapic solution added. This was diluted in an appropriate amount of sterile distilled water, fortified with 1 mg/kg imazapic, and placed in a sterilized incubator (GXZ-300B, Jiangnan Instrument Manufacture, Ningbo, P.R. China). The test variables were as follows: three temperatures (15, 25, and 35°C), four soil moisture contents (15, 40, 60, and 90%), (W water/W soil), and three soil pH values (6.0, 7.0, and 8.0). Chicken manure was added at the levels of 21.0, 90.7, and 160.5 g to 1000 g of dry soil soil and the final organic matter content of the soils was 0.55, 1.0, 2.5, and 4.0%, The soil sample with an organic matter content of 0.55% was a control, without the addition of chicken manure. The biogas slurry consisted of 0.9% and 3.6% soil by weight. The degradation of imazapic were also tested in sterilized and unsterilized soil samples. The sterilized soil was prepared by autoclaving under 121°C for 1 h for three consecutive days. For experiments with sterilized soil, all of the equipment used was autoclaved. Most treatments in the study were incubated at 25°C, 60% moisture content, and in darkness, with the exceptions being temperature and moisture, where soils were incubated under an assigned temperature and moisture. There were three replicates per treatment. During the experiment, to maintain soil moisture as assigned for each treatment, samples were weighed and sterile distilled water added as necessary. Soil samples of 10 g (dry weight equivalent) were taken for analysis from each treatment on days 0, 1, 3, 7, 14, 21, 30, 45, 60, 90, 120, and 150. The samples were placed in plastic bags and stored at −18°C until analysis.

#### Sample extraction

Each samples was mixed with 20 mL NaOH solution (0.5 mol/L) in 50-mL centrifuge tubes. The mixtures were shaken mechanically for 1 h and centrifuged at RCF 1615 × g (3800 r/min) for 5 min. Then, 10 mL of the supernatant was transferred into a 200-mL separatory funnel, and the supernatant pH value was adjusted to 1.8–2.0 by adding HCl (7 mol/L). The organic phase was collected after three successive extractions using 20 mL of trichloromethane, and the combined organic phase was concentrated by a rotary evaporator at 40°C. The residue was dissolved in methanol, with a final volume of 2 mL, filtered through 0.22-μm syringe filters, and transferred into an auto-sampler vial for analysis.

#### Analytical method

The amount of imazapic was determined using high performance liquid chromatography-tandem mass spectrometry (HPLC-MS/MS). The analyses were performed on an Agilent 1200 HPLC and an Agilent 6410B triple quadrupole mass spectrometer, equipped with an electrospray ionization interface source and operating in positive mode (Agilent Technologies, Santa Clara, CA, USA). A reverse phase “eclipse plus” C18 (2.1 × 50 mm, 3.5 μm) column from Agilent Technologies was used at 30°C. The mobile phase was a 0.1% methanol—formic acid solution (60:40, v:v) at a flow rate of 0.2 mL/min. The injection volume in the HPLC system was 5 μL. The MS detection conditions were: desolvation gas flow rate, 8.0 L/min; desolvation gas temperature, 350°C; nebulizer gas (N_2_) pressure, 241.3 kPa; capillary voltage 4000 V; MS 1 and MS 2 heater temperature, 100°C.

The soil used in experiments Was confirmed to contain no imazapic. A series of preliminary studies validated the imazapic recovery procedure. The recovery study was conducted in soil spiked at three different imazapic concentrations: 0.02, 0.2, and 0.5 mg/kg. The recoveries were from 83.3 to 102.4%, and the relative standard deviations were 1.3–10.2%. The limit of quantification of imazapic in soil was 0.003 mg/kg. The results showed that the method was suitable and repeatable for the analysis of imazapic residues in soil.

### Data analysis

Degradation of imazapic in soil was described by first-order kinetics. The half-life was calculated by fitting first-order equations to observe the degradation patterns as: *Ct* = *Co*e^−*kt*^, where C_t_ is the imazapic concentration at time t (d) (mg/kg), C_o_ is the initial concentration of imazapic in soil (mg/kg), and k is the first-order rate constant (d^−1^). The t_1/2_ was calculated from ln2/k. All experiments were conducted in triplicate, and all values were reported as means with their standard deviation. Microsoft Excel 2010 and SPSS (Version 18.0, SPSS, Chicago, IL, USA) and Duncan’s method were used for statistical tests (P≤0.05). There was no significant difference between replications (P≤0.05).

## Results and discussion

### Adsorption studies

**Fig 2:**
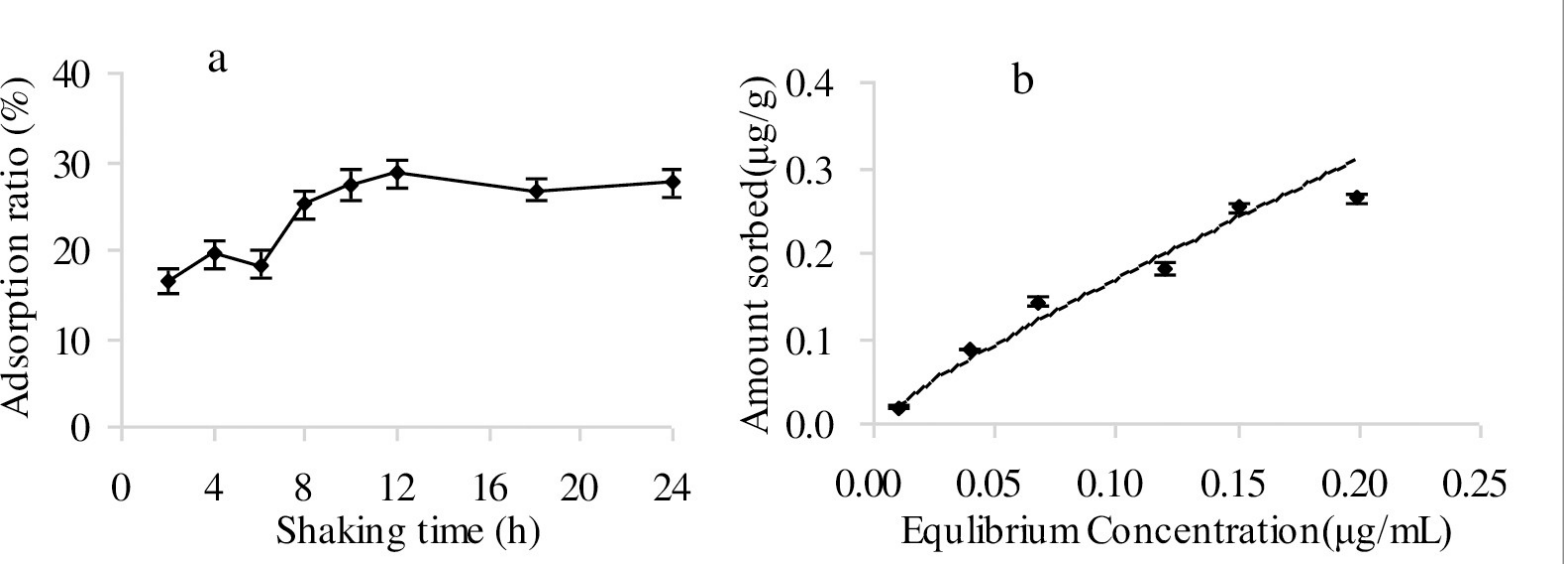
Imazapic adsorption ratio under (a) different shaking times, (b) adsorption isotherms of imazapic. Each data point in the figure is the mean of three replicates. Error bars represent the SD of the mean.

In the adsorption experiments, the analysis of the control sample showed that the concentration of imazapic did not change during 24 h of shaking. The adsorption equilibration time was found to be 12 h and was used in adsorption studies. When equilibrium was reached, the extension of the shaking time had no effect on adsorption (Fig 2A). Adsorption of imazapic was well described by the Freundlich equation (Fig 2B), K_f_ = 1.2602, 1/n = 0.8712, r^2^ = 0.9780. The constant 1/n indicates the degree of nonlinearity between solution concentration and adsorption. It was found that the adsorption isotherm of imazapic was an L–type with 1/n < 1. This type of isotherm is characterized by a decreasing slope as the concentration increases due to the decrease in vacant sorption sites as the adsorbent becomes covered. K_f_ is a measure of the degree or strength of adsorption. The capacity for adsorption was positively correlated with K_f_. This was consistent with previous research [18]. The result indicated that the soil had a stronger adsorption capacity for imazapic. Soil texture plays an important role in soil adsorption. The smaller the soil particles the greater the surface area of the soil, and soil adsorption capacity increases [19]. The clay, slit, and sand contents were 21.8, 69.1, and 9.1%, respectively. The clay and slit contents were greater than the sand content, which favored a high soil adsorption capacity. Moreover, within the soil mineral composition there was an 11% vermiculite content. Vermiculite is a clay mineral that enhances soil adsorption [20].

### Degradation experiment

#### Effect of temperature on imazapic degradation

**Fig 3:**
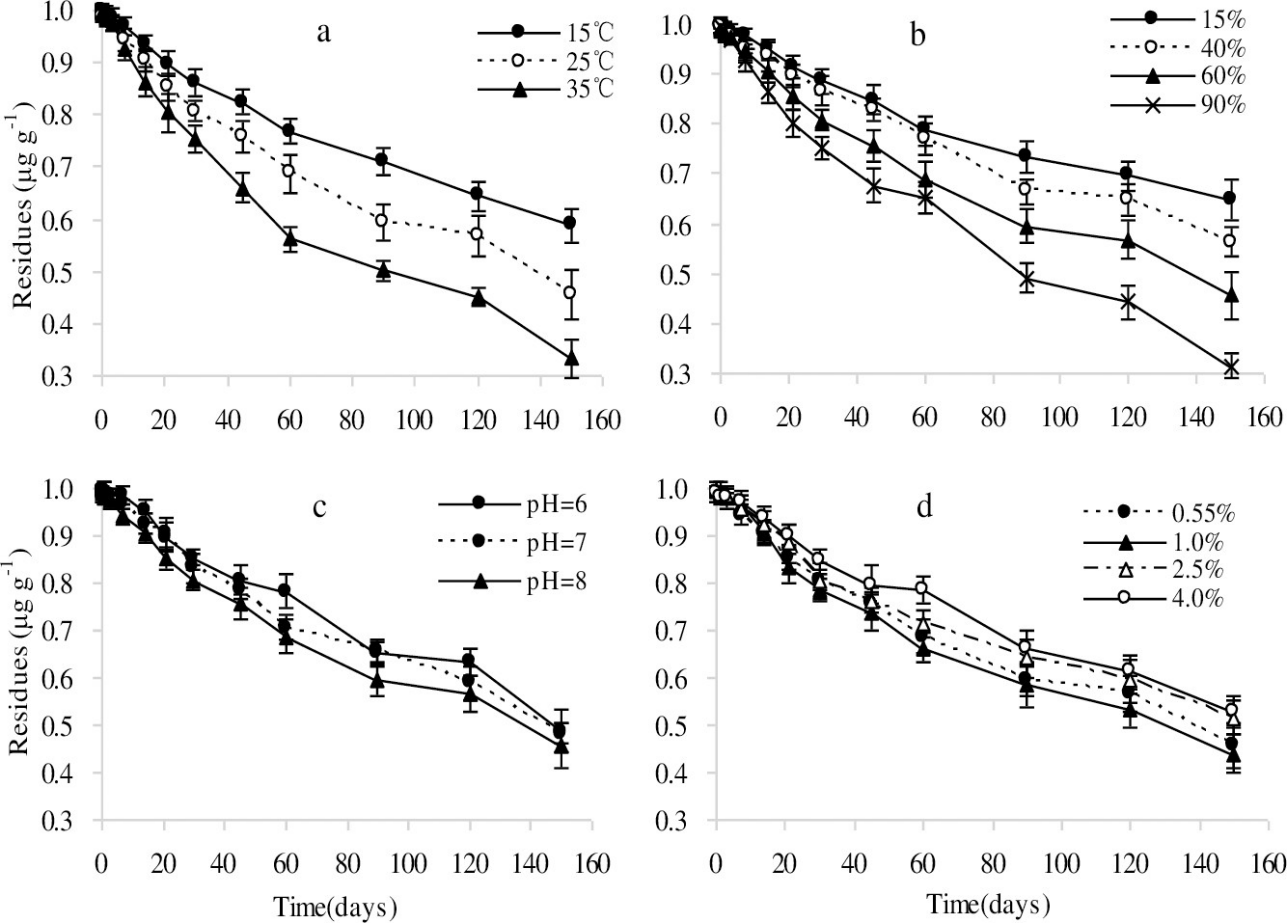
Degradation kinetics of imazapic under different (a) temperatures, (b) moisture contents, (c) pH values, and (d) organic matter contents. Each data point in the figure is the mean of three replicates. Error bars represent the SD of the mean.

We studied the degradation of imazapic at three temperatures in incubators under laboratory conditions. The results indicated that the imazapic degradation rate increased as temperature increased (Fig 3A and Table 3). Imazapic was detected up to day 150. It was found that imazapic degradation was most rapid at 35°C (half-life, t_1/2_ = 99.0 d), followed by 25°C (t_1/2_ = 138.6 d), and 15°C (t_1/2_ = 192.5 d). These results were consistent with those reported previously for the imidazolinone herbicides imazapyr and imazaquin [15,21], and other herbicides, such as cloransulam-methy and florasulam [22,23], for which degradation increases as temperature increases. The results indicate that imazapic will degrade rapidly in summer at high temperatures. A previous study also found that high temperatures will decrease equilibrium adsorption [24]. Any pesticide that is not adsorbed will be easily available to microorganisms. Imazapic degradation was primarily a microbial process. High temperatures (suitable for microbial growth) will increase the number of microbes present and enhance microbial activity [25, 26]. The number of microbes and their activity play an important role in the process of biodegradation.

**Table 3:** Half-lives and first-order equations of imazapic in response to different factors.

Factors		Degradation equation	R^2^	Initial concentration	Standard error	*t*_1/2_ (d)	Standard error
Temperature	15°C	C = 0.9827e^-0.0036t^	0.9899	0.9988	0.0068	192.5	0.6279
	25°C	C = 0.9668e^-0.0050t^	0.9844	0.9885	0.0121	138.6	4.7495
	35°C	C = 0.9562e^-0.0070t^	0.9783	0.9965	0.0114	99.0	4.3978
Soil moisture content	15%	C = 0.9822e^-0.0030t^	0.9805	0.9935	0.0031	231.0	7.7274
	40%	C = 0.9788e^-0.0038t^	0.9871	0.9976	0.0082	182.4	8.1025
	60%	C = 0.9668e^-0.0050t^	0.9844	0.9885	0.0121	138.6	4.9301
	90%	C = 0.9698e^-0.0072t^	0.9885	0.9968	0.0088	96.3	3.7829
Soil pH value	6	C = 0.9989e^-0.0045t^	0.9801	0.9844	0.0070	154.0	8.1197
	7	C = 0.9850e^-0.0047t^	0.9881	0.9939	0.0052	147.4	0.0282
	8	C = 0.9668e^-0.0050t^	0.9844	0.9885	0.0121	138.6	4.9301
Organic matter content	0.55%	C = 0.9668e^-0.0050t^	0.9844	0.9885	0.0121	138.6	4.9301
	1.0%	C = 0.9701e^-0.0054t^	0.9821	0.9946	0.0056	128.3	8.1037
	2.5%	C = 0.9702e^-0.0044t^	0.9772	0.9969	0.0177	157.5	10.4909
	4.0%	C = 0.9852e^-0.0042t^	0.9927	0.9896	0.0098	165.0	9.5692
Biogas slurry content	0	C = 0.9668e^-0.0050t^	0.9844	0.9885	0.0121	138.6	4.9301
	0.9%	C = 0.9654e^-0.0057t^	0.9801	0.9892	0.0059	121.6	5.7683
	3.6%	C = 0.9680e^-0.0069t^	0.9853	0.9988	0.0117	100.4	2.1853
Microorganisms	Unsterilized	C = 0.9668e^-0.0050t^	0.9844	0.9885	0.0121	138.6	2.6102
	Sterilized	C = 1.0101e^-0.0019t^	0.9811	1.0369	0.0089	364.7	2.5757

#### Effect of soil moisture on imazapic degradation

The influence of soil moisture on imazapic degradation is shown in Fig 3B. Imazapic degradation was fastest at a soil moisture content of 90%, with a t_1/2_ of 96.3 d. The t_1/2_ values of imazapic at a soil moisture content of 15, 40, and 60% were 231.0, 182.4, and 138.6 d, respectively (Table 3). This indicates that imazapic would have a long residual period when applied in drought prone areas, and appropriate irrigation could promote its degradation. The degradation of the imidazolinone herbicides imazapyr and imazaquin is similarly affected by soil moisture, with degradation occurring faster at a higher soil moisture contents [15,21]. The proliferation of microorganisms was directly affected by soil moisture [15]. A high soil moisture, with high levels of microbial activity, would stimulate the microbial degradation of imazapic [22,27,28]. Moreover, pesticide adsorption in soil was greater at a low moisture content [29].

#### Effect of soil pH on imazapic degradation

Soil pH has a significant influence on the behavior of imidazolinone herbicides in soil [5]. Of the three soil pH values examined, alkaline soil (pH 8.0) resulted in the fastest degradation rate of imazapic (t_1/2_ = 138.6 d), neutral soil (pH 7.0) produced a slower degradation rate (t_1/2_ = 147.4 d), and acid soil (pH 6.0) produced the slowest degradation rate (t_1/2_ = 154.0 d) (Fig 3C, Table 3). The result indicates that imazapic would have a long residual period in acidic soil. The adsorption of the imidazolinone herbicides imazamox and imazethapyr has been reported to decrease when soil pH becomes more alkaline [12,15,30,31]. Imazapic adsorption is similarly affected. The more imazapic that is adsorbed, the less it can be degraded by microorganisms [32]. This may be one reason why alkaline soil conditions would favor imazapic degradation. Imazapic is a weak acid compound, and predominantly exists in an anionic form in alkaline soils [18]. The surface of soil particles tends to be negatively charged as the soil pH increases, and the repulsion of the anionic form by the negatively charged surface explains why imazapic adsorption is inhibited at the soil surface. Moreover, the simple structure is more easily decomposed by microorganisms [33].

#### Effect of soil organic matter content on imazapic degradation

Soil organic matter content has an influence on microbial activity and soil adsorption. The experiment investigated the degradation of imazapic in soils with different organic matter contents (Fig 3D). The results showed that imazapic was degraded faster in soils with a low organic matter content than in soils with a high organic matter content. The degradation was most rapid in soil with 1.0% organic matter content (t_1/2_ = 128.3 d). The next most rapid degradation was in soil with 0.55% organic matter content (t_1/2_ = 138.6 d). Degradation was slower in the soil with 2.5% organic matter content (t_1/2_ = 157.5 d), and slowest in the soil with 4% organic matter content (t_1/2_ = 165.0 d) (Table 3). The influence of soil organic matter content on imazapic degradation was the combined result of its effects on microorganisms and soil adsorption. Chicken litter used as an organic amendment can promote the growth of microorganisms and accelerate the degradation of imazaquin [34, 35]. Organic matter also affects the ability of soil to adsorb organic compounds [23]. The degradation and adsorption of organic compounds are coupled processes, with adsorption being unfavorable for microbial degradation. Adsorbed imazapic is difficult for microorganisms to degrade. Krauss and Wilcke have reported that soil adsorption was positively correlated with soil organic matter content [36]. Imazethapy has been reported to degrade slowly in soils with a large organic matter content, which provides a greater adsorptive potential [37].

#### Imazapic degradation in sterilized and unsterilized soil

**Fig 4:**
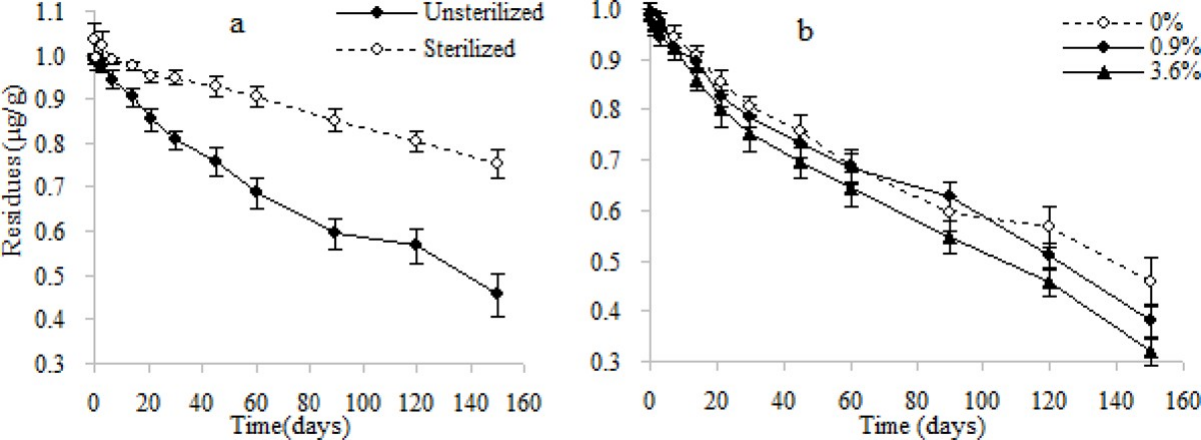
Degradation kinetics of imazapic (a) under the influence of microorganisms, (b) with the application of biogas slurries. Each data point in the figure is the mean of three replicates. Error bars represent the SD of the mean.

It has been reported that microbial activity significantly affects the degradation of imidazolinone herbicides in soil [15]. This study evaluated the influence of microorganisms on imazapic degradation in sterilized and unsterilized soil (Fig 4A). On day 60, only 12.5% of imazapic residues were dissipated in sterilized soil compared to the 30.5% dissipation rate recorded in unsterilized soil. Imazapic degraded more slowly in sterilized soil (t_1/2_ = 364.7 d) than in unsterilized soil (t_1/2_ = 138.6 d) (Table 3). The results indicated that imazapic degradation was primarily a microbial process. This is similar to imidazolinone herbicides, such as imazapyr, for which degradation is primarily a microbial process [21,38,39]. In the process of microbial degradation, microbial biomass and its activity significantly affected the process. Soil sterilization can reduce the microbial biomass and activity of the soil. A similar trend has been reported for thifensulfuron and mesotrione, with their half-lives being significantly longer in sterilized soils than in unsterilized soils, indicating that their degradation is primarily a microbial process [40,41].

#### Effect of biogas slurries on imazapic degradation

In this study, the process of imazapic degradation was investigated under the addition of two different biogas slurry applications (Fig 4B). Degradation was fastest in soil with the addition of 3.6% biogas slurry (t_1/2_ = 100.4 d), followed by the addition of 0.9% biogas slurry (t_1/2_ = 121.6 d). Imazapic degradation was slowest in soil without biogas slurry (t_1/2_ = 138.6 d) (Table 3). The results showed that the addition of biogas slurry can accelerate imazapic degradation. Biogas slurry has been confirmed to contain major and trace elements, and organic matter, and can be beneficial to microbial growth in soil [42]. Biogas slurry can increase the number of species present and the overall quantity of microorganisms, leading to a faster microbial degradation of imazapic. The soil moisture and organic matter content increased as biogas slurry was added, with higher soil moisture and organic matter contents favoring biodegradation in soil. It has been reported that the use of biogas slurry as a soil amendment accelerates the degradation of other herbicides [23,43].

## Conclusions

It was found that imazapic has a long residual period in soil and microbial degradation was the primary mechanism of imazapic degradation. The microbial degradation of imidazolinone herbicides is correlated with soil properties and adsorption [31]. The results of this study indicate that soil has a certain adsorption capacity for imazapic. Imazapic degradation increased as the soil pH increased. Temperature and moisture are critical variables affecting imazapic degradation in soil. High temperatures coupled with moist soil conditions favor microbial activity and accelerate imazapic degradation. Imazapic degraded faster in soils with a low organic matter content. Furthermore, adding biogas slurry reduced the half-life of imazapic in soil, and therefore biogas slurry could be used as a soil amendment. Imazapic residues could be a serious problem in drought and acidic areas. The results of this study can be applied to accelerate imazapic degradation in the field. Further studies are needed to understand the adsorption of imazapic under different conditions.

## References

[1] Matocha MA, Vencill WK. The persistence of imazapic in peanut (Arachis hypogaea) crop rotations, Weed Technol. 2003, 17, 325–329.

[2] Grichar WJ, Dotray PA, Baughman TA. Influence of simulated imazapic and imazethapyr herbicide carryover on cotton (gossypium hirsutum L.), International Journal of Agronomy. 2012,1687–8159.

[3] Grey TL, Prostko EP, Bednarz CW, Davis JW. Cotton (Gossypium hirsutum) response to simulated imazapic residues, Weed Technol. 2013, 19, 1045–1049.

[4] York AC, Jordan DL, Batts RB, Culpepper AS. Cotton response to imazapic and imazethapyr applied to a preceding peanut crop. J Cotton Sci. 2000, 3, 210–216.

[5] Ulbrich AV, Souza JRP, Shaner D. Persistence and carryover effect of imazapic and imazapyr in brazilian cropping systems1.Weed Technology, 2005, 19, 986–991.

[6] National Bureau of Statistics of China, http://www.stats.gov.cn/tjsj/ndsj/2016/indexch.htm.

[7] Dong W, Tang F, Zhang X. Present situation and development Suggestions of peanut industry in henan province. Henan agricultural science, 2007(10):8–10+15.

[8] Zhao S, Ye F. Applications of imidazolinone herbicide and its degradation, Plant Protection. 2009, 35, 15–19.

[9] Su W, Sun L, Wu R, Ma Y, Wang H, Xu H, Yan Z, Lu C. Effect of imazapic residues on photosynthetic traits and chlorophyll fluorescence of maize seedlings, Photosynthetica. 2017, 55:294–300.

[10] Su W, Sun L, Zhang Q, Wu R, Wang H, Lu C, Zhang Y. Effects of imazapic residues on the growth and photosynthetic parameters of wheat seedlings as succeeding crop, J. Triticeae Crops. 2013, 33, 1226–1231.

[11] Su W, Sun, Wu R, Wang H, Ma Y, Gao X, Zhang Y, Lu C. Sensitivity of succeeding crops to simulated imazapic residue in laboratory, Agrochemicals. 2014, 53, 260–262.

[12] Marchesan E, Dos Santos FM, Grohs M, De Avila LA, Machado SL, Senseman SA, Massoni PF, Sartori GM. Carryover of imazethapyr and imazapic to nontolerant rice, Weed Technol. 2010, 24, 6–10.

[13] Prostko EP, Grey TL, Morgan RN, Davis JW. Oat (Avena sativa) response to imazapic residues, Weed Technol. 2005, 19, 875–878.

[14] Jordan DL, Lancaster SH, Lanier JE, Lassiter BR, Johnson PD. Weed management in peanut with herbicide combinations containing imazapic and other pesticides, Weed Technol. 2009, 23, 6–10.

[15] Chen X, Ge B, Chang C. Advances in studies on the environmental behaviors of imidazolinone herbicides, Fine Chemical Intermediates. 2010, 40, 1–6.

[16] Wei K, Ma G. The soil of Henan province. Beijing, China Agriculture Press, 2004.

[17] Chemicals-adsorption-desorption using a batch equilibrium method: GB/T 21851–2008[S]. China Standards Press, Beijing.

[18] Gao C. The toxicity of midazolinone herbicides to microorganisms and earthworm and its environmental behaviors, Guangxi University Nanning, 2013.

[19] Zhang S, Zhao B, Wang F, Wan J, Lin Y, Zhou Y, Zhou L. Effects of soil particles on their adsorption performance of chloredane and their acute toxicity. Chinese Journal of Environmental Engineering, 2017,11(06):3839–3845.

[20] Tan R, Zhu J, Wang P, Yang Y, Zhu Y, Jin Q. Adsorption study of vermiculite, montmorillonite and bentonite on heavy metal chromium.China powder science and technology, 2017, 23(06):82–89.

[21] Wang X, Wang H, Fan D. Degradation and metabolism of imazapyr by microorganisms in soils, Research of Environmental Sciences. 2004, 17, 42–45.

[22] Cupples AM, Sims GK, Hultgren RP, Hart SE. Effect of soil conditions on the degradation of cloransulam-methyl. J Environ Qual. 2000, 29(3):786–794.

[23] Su W, Xu H, Hao H, Wu R, Wang H, Lu C. Effect of environmental conditions on the degradation of florasulam in typical soils of northern china. Journal of Environmental Quality, 2017, 46(3), 553 10.2134/jeq2016.11.0449 28724091

[24] Hulscher TEMT, Comelissen G. Effect of temperature on sorption equilibrium and sorption kinetics of organic micropollutants—a review, Chemosphere. 1996, 32, 609–626.

[25] Madani ME, Azzouzi ME, Zrineh A, Martens D, Kettrup A. pH effect and kinetic studies of the binding behaviour of imazethapyr herbicide on some Moroccan soils, Fresenius Environ. Bull. 2003, 12, 1114–1119.

[26] Kurola J, Salkinoja-Salonen M. Potential for biodegradation of anthropogenic organic compounds at low temperature in boreal soils, Soil Biol Biochem. 2007, 39, 1206–1212.

[27] Hultgren RP, Hudson RJ, Sims GK. Effects of soil pH and soil water content on prosulfuron dissipation, J Agric Food Chem. 2002, 50, 3236–3243. 12009993 10.1021/jf011477c

[28] Bundt AC, Avila LA, Pivetta A, Agostinetto D, Dick DP, Burauel P. Imidazolinone degradation in soil in response to application history, Planta Daninha. 2015, 33, 341–349.

[29] Shelton DR, Parkin TB. Effect of moisture on sorption and biodegradation of carbofuran in soil, J. Agric. Food Chem. 1991, 39, 2063–2068.

[30] Bresnahan GA, Koskinen WC, Dexter AG, Lueschen WE. Influence of soil pH-sorption interactions on imazethapyr carry-over. Journal of Agricultural & Food Chemistry, 2000, 48(5), 1929–1934. 10820117 10.1021/jf990543w

[31] Aichele TM, and Penner D. Adsorption, desorption, and degradation of imidazolinones in soil. Weed Technology, 2005, 19(1), 154–159.

[32] Szmigielski A, Geisel B, Holm F, Johnson E, Schoenau J. Application of a Laboratory Bioassay for Assessment of Bioactivity of ALS-Inhibiting Herbicides in Soil. Herbicides and Environment, InTech, 2011,p. 217–228.

[33] You M, Liu X. Biodegradation and bioremediation of pesticide pollution. Chinese Journal of Ecology. 2004,23 (1):73–77.

[34] Gomez E, Ferreras L, Toresani S. Soil bacterial functional diversity as influenced by organic amendment application. Bioresource Technology, 2006, 97(13), 1484–1489. 10.1016/j.biortech.2005.06.021 16168637

[35] Wang H, Li Y, Wei G, Wang X. Imazaquin detradation and metabolism in a sandy loam soil amended with farm litters. Journal of Environmental Sciences, 2007, 19:1108–1113. 10.1016/s1001-0742(07)60180-6 17966517

[36] Krauss M, Wilcke W. Sorption strength of persistent organic pollutants in particle-size fractions of urban soils, Soil Sci Soc Am J. 2002, 66, 430–437.

[37] Goetz AJ, Lavy TL, Gbur EEJ. Degradation and field persistence of imazethapyr. Weed Science, 1990, 38(4/5), 421–428.

[38] Alister C, Kogan M. Efficacy of imidazolinone herbicides applied to imidazolinone-resistant maize and their carryover effect on rotational crops. Crop Prot. 2005, 24:375–379.

[39] Chen T. Study on Analytieal Method and Environmental Behavior of Imazethapyr Herbieide in Soil, Shandong university Shandong, 2007.

[40] Tang M, Guo Z. Degradation of thifensulfuron in brown earth, Jecol Rural Environ. 2010, 26, 185–188. 37

[41] Quan G, Yin C, Chen T, Yan J. Degradation of Herbicide Mesotrione in Three soils with Differing Physicochemical Properties from China. J Environ Qual. 2015, (5):1631–1637. 10.2134/jeq2014.12.0528 26436279

[42] Ju P. Effects of radish vegetal specialties and quality and soil characteristics with biogas slurry. Northwest A & F University, Yangling, 2008.

[43] Kadian N, Gupta A, Satya S, Mehta RK, Malik A. Biodegradation of herbicide (atrazine) in contaminated soil using various bioprocessed materials, Bioresource Technol. 2008, 99, 4642–4647. 10.1016/j.biortech.2007.06.064 17826992

